# Implementing AI-enabled chest X-ray for community-based integrated screening for tuberculosis, chronic respiratory diseases, and cardiovascular diseases in Nigeria

**DOI:** 10.1186/s44263-026-00313-7

**Published:** 2026-08-05

**Authors:** Chidimma Okoye, Jude Ilozumba, John Oko, Amana Effiong, Young Oluokun, Chinwe Eze, Obioma Akaniro, Emperor Ubochioma, Collins Nwachi Ugwu, Rena Fukunaga, Toufiq Rahman, John Bimba, Jacob Creswell, Chukwuebuka Ugwu

**Affiliations:** 1 TB/HIV Department, Catholic Caritas Foundation of Nigeria, Abuja, Nigeria; 2 PRIME Data Nexus, Enugu, Nigeria; 3 National Tuberculosis and Leprosy Control Programme, Abuja, Nigeria; 4 Department of Internal Medicine, Alex Ekwueme Federal University Teaching Hospital Abakaliki, Abakaliki, Ebonyi State Nigeria; 5 Centers for Disease Control and Prevention, Atlanta, GA USA; 6 Stop TB Partnership, TB REACH, Geneva, Switzerland; 7 Zankli Research Centre, Bingham University, Nasarawa, Nigeria; 8 Centre for Tuberculosis Research, Liverpool School of Tropical Medicine, Liverpool, UK

**Keywords:** Tuberculosis, Artificial intelligence, Chest radiography, Community-based screening, Active case finding, Cardiovascular disease, Chronic respiratory disease, Non-communicable diseases, Nigeria

## Abstract

**Background:**

Low- and middle-income countries face a growing dual burden of communicable and non-communicable diseases, while services remain vertically organised. In Nigeria, tuberculosis (TB) services are established at primary care level, whereas access to cardiovascular disease (CVD) and chronic respiratory disease (CRD) care remains limited in rural settings. Artificial intelligence (AI) enabled chest X-ray can integrate TB screening with identification of other cardiopulmonary abnormalities at community level. We describe the screening outcomes and referral cascade of a community-based, AI-enabled integrated programme.

**Methods:**

We conducted a non-randomised descriptive study using routinely collected programme data from five Local Government Areas in Ebonyi and Nasarawa States, January 2023 to December 2024. Community outreach used portable digital chest X-ray with AI software to screen individuals aged six years and above. People with presumptive TB underwent Xpert MTB/RIF (Mycobacterium tuberculosis/rifampicin) testing on the GeneXpert platform, while non-TB radiographic abnormalities were referred for further evaluation. Descriptive analyses summarised screening yield, diagnostic outcomes, and linkage to care.

**Results:**

In total, 9,585 individuals were screened through 93 outreach activities, and 3,166 (33%) chest radiographs were flagged as abnormal by AI. Overall, 1,336 were classified as having presumptive TB, of whom 1,123 (84%) produced sputum for Xpert MTB/RIF testing. 204 were diagnosed with bacteriologically confirmed TB, and 194 (95%) were initiated on treatment. A further 199 people were clinically diagnosed with TB following radiologist and/or clinical review. Among abnormal radiographs, 2,367 (75%) showed features suggestive of CVDs or CRDs. All such individuals were referred to tertiary facilities; however, only 12% completed the referral. Programmatic adaptations supported TB linkage but had limited impact on non-TB referral completion.

**Conclusions:**

AI-enabled community chest X-ray screening is feasible for TB case finding in rural Nigeria and achieves high linkage to TB treatment. Chest radiography also identified many abnormalities requiring further evaluation for CVD and CRD, most not confirmed within the study. Limited decentralisation of non-communicable disease services constrains care continuity for CVDs and CRDs. Integrated screening programmes should be paired with strengthened primary healthcare capacity, complementary tools such as blood pressure measurement, and context-specific community engagement strategies.

## Background

Low- and middle-income countries (LMICs) are experiencing a growing double burden of disease, in which communicable diseases coexist with an increasing prevalence of non-communicable diseases (NCDs) within the same population [1]. This reflects overlapping demographic and epidemiological transitions, including population growth, increased life expectancy, urbanisation, and lifestyle changes [2]. In parallel, many LMICs face a double burden of malnutrition, with undernutrition and overnutrition occurring concurrently, further increasing vulnerability to both infectious and chronic conditions [3, 4]. These converging trends place sustained pressure on health systems that remain largely organised around vertical, disease-specific programmes.

Nigeria exemplifies this challenge, bearing the highest tuberculosis (TB) burden in Africa, alongside persistent gaps in the prevention and management of drug-resistant TB and TB/HIV co-infection [5]. TB remains among the leading causes of death nationally. The World Health Organization (WHO) estimated approximately 56,000 TB deaths among HIV-negative individuals in Nigeria in 2024, with an additional 5,800 deaths among people living with HIV [5]. At the same time, the burden of NCDs has increased steadily. In 2019, NCDs accounted for approximately 29% of all deaths in Nigeria [6]. Cardiovascular diseases contributed about 11% of all deaths, while CRDs accounted for approximately 2%, alongside a substantial loss of disability-adjusted life years [6].

Hypertension, the leading modifiable risk factor for cardiovascular disease, affects over 30% of Nigerian adults and contributes substantially to myocardial infarction and stroke [7]. National evidence also indicates a high prevalence of additional CVD risk factors, including overweight and obesity, diabetes mellitus, physical inactivity, tobacco use, exposure to household air pollution, and unhealthy dietary patterns [6, 8]. Hospital-based studies further demonstrate a sustained rise in CVD-related admissions and mortality. A 15-year review from Lagos reported that CVDs accounted for 20.8% of all medical admissions and 30.4% of in-hospital mortality, underscoring the growing clinical and economic burden of chronic diseases in Nigeria [9].

Nigeria’s primary healthcare system is relatively better resourced for the surveillance and management of endemic communicable diseases such as malaria, TB, and HIV, largely through established vertical programmes and sustained donor support [5, 10]. While TB case detection and treatment coverage improved between 2019 and 2023 [5], access to NCD prevention, diagnosis, and long-term care remains limited, particularly at the primary healthcare level. For example, a recent assessment of primary healthcare facility readiness in Nigeria found that many facilities lacked essential diagnostic tools, trained personnel, and medicines required to implement the basic WHO Package of Essential NCD Interventions (PEN), limiting routine screening and management of conditions such as hypertension and diabetes [10–12]. Services for CVDs and CRDs are often unavailable or fragmented at facilities closest to communities, resulting in delayed diagnoses, high out-of-pocket expenditure, and poor continuity of care. These gaps undermine progress toward universal health coverage and disproportionately affect rural and hard-to-reach populations [11].

Integrated, community-based approaches that combine screening for communicable and non-communicable diseases are therefore increasingly important. Systematic chest X-ray-based screening has re-emerged as an effective strategy for TB case finding in high-burden settings [13, 14]. AI-enabled chest X-ray interpretation systems have demonstrated high accuracy for TB screening and increasing utility in identifying non-TB abnormalities, while reducing reliance on scarce specialist expertise [15–17]. In 2021, WHO endorsed the use of AI-based chest X-ray interpretation for TB screening, recognising its potential to expand access, improve efficiency, and strengthen case detection in resource-limited settings [18]. The role of point-of-care imaging, including portable chest radiography, in enabling early, accurate diagnosis of cardiovascular and respiratory diseases in low-resource settings is increasingly recognised as a paradigm shift in diagnostic access [19]. Evidence also shows that chest radiography can identify abnormalities suggestive of CRDs and CVDs, including cardiomegaly and chronic lung changes [20].

The INTEGRATE-TB project (Technology Assisted Integrated Health Care Services and Community TB Preventive Therapy Expansion), a community-based active case-finding project delivered under the TB REACH Wave 10 initiative, was designed to apply community-based, AI-enabled chest X-ray screening for tuberculosis, while identifying radiographic abnormalities suggestive of cardiovascular and chronic respiratory diseases in remote rural communities in Nigeria. By using TB screening as an entry point for broader lung and cardiovascular health assessment, the project aimed to expand access to diagnostic services for underserved populations and to generate operational evidence within routine programme settings. This paper describes the screening outcomes and referral cascade, and highlights key operational considerations, guided by the Template for Intervention Description and Replication (TIDieR) framework [21].

## Methods

### Study design and setting

This was a non-randomised descriptive study of a community-based AI-enabled screening programme, using routinely collected programme data. It was conducted across five Local Government Areas (LGAs) in Nigeria: three LGAs in Ebonyi State (South-East) and two LGAs in Nasarawa State (North-Central), between January 2023 and December 2024.

The two study states have distinct demographic and epidemiological profiles that contextualise the rationale for targeting these communities for active case finding.

Ebonyi State is located in South-East Nigeria, with a population of 3,240,022, of whom approximately 95% are rural dwellers [22]. An estimated 240,192 residents live in communities that are hard-to-reach by healthcare services, and a further 57,155 live in informal settlements. In 2021, Ebonyi State notified 1,472 people with TB [23], representing a case notification rate of 42 per 100,000 population. Nasarawa State is in North-Central Nigeria, with a population of 2,990,009 [22]. Approximately 30% of the population resides in rapidly urbanising areas contiguous with the Federal Capital Territory (Abuja), while 308,237 residents live in hard-to-reach communities. In 2021, Nasarawa State notified 4,261 people with TB, a case notification rate of 142 per 100,000 population [23].

Within both states, all the selected LGAs (three in Ebonyi: Ikwo, Ishielu, and Izzi; and two in Nasarawa: Karu and Lafia) had notification rates below their respective state averages based on 2021 data from the State TB and Leprosy Control Programme reports, except Lafia [23]. LGA-level notification rates were as follows: Ikwo 32 per 100,000, Ishielu 31 per 100,000, Izzi 39 per 100,000, Karu 104 per 100,000, and Lafia 176 per 100,000. Many communities within all five LGAs are classified as hard-to-reach, which may contribute to low case notification rates. This justifies prioritising them for systematic community-based active case finding, in line with WHO guidance on targeting screening interventions toward populations with persistently low detection [24, 25]. Although Lafia LGA had a notification rate above the Nasarawa State average, it was selected because a significant proportion of its communities are classified as hard-to-reach by healthcare services, with limited transport connectivity and poor access to diagnostic facilities, meaning that the state-level notification rate does not fully reflect the detection gap within these underserved communities.

The three selected LGAs in Ebonyi (Ikwo, Ishielu, and Izzi) also have a notable presence of illegal artisanal quarrying and mining activities, with occupational dust exposure from these sites recognised as a risk factor for chronic respiratory disease, likely contributing to the higher burden of CRD-suggestive abnormalities observed in Ebonyi communities.

In both states, primary healthcare facilities had access to the GeneXpert testing platform and to directly observed treatment (DOTS) services at the time of implementation, consistent with national programme standards. However, access to chest radiography, specialist cardiovascular services, and respiratory diagnostics at the primary care level was limited, and these services were generally only available at tertiary referral facilities.

### Screening programme description

This study reported a community-based TB screening programme that used AI-enabled chest X-ray to simultaneously screen for tuberculosis and identify radiographic abnormalities suggestive of CVDs and CRDs. The programme used portable digital chest X-ray (PDX) machines equipped with AI software to enable real-time interpretation during outreach activities, in line with WHO recommendations on systematic TB screening and use of chest radiography.

Tuberculosis screening served as the primary entry point, with non-TB abnormalities identified and referred through established pathways. The programme aimed to improve early detection, strengthen linkage to care, and generate operational evidence on the feasibility of AI-enabled, multi-disease screening in underserved settings [24, 26].

### Community engagement and delivery

Prior to implementation, structured engagement was conducted with community leaders, women’s and youth groups, religious institutions, and local government health teams to introduce the programme and adapt implementation plans to local contexts, consistent with WHO recommendations for community-based TB screening [18, 25]. Outreach schedules were aligned with community calendars, including market days and cultural events.

Community volunteers were recruited from respected community members to support mobilisation, health education, and participant flow. Health education sessions were delivered in local languages using visual aids and public address systems, following best practices for community TB care and prevention [18, 25].

### Outreach implementation and eligibility

Outreach teams comprised a radiographer, a linkage coordinator, the Local Government TB and Leprosy Supervisor (LGTBLS), community volunteers, an administrative assistant responsible for real-time data entry, and Catholic Caritas Foundation of Nigeria (CCFN) supervisory staff. Outreach activities were conducted in accessible public spaces such as markets, town halls, and school compounds.

Individuals aged six years and above were eligible for screening, excluding pregnant women. Verbal informed consent was obtained from adult participants, and parental consent was obtained for minors. Following consent, participants underwent symptom screening and chest radiography, with AI-generated results available within an average of 3 min.

### Screening tools and procedures

Portable digital chest X-ray machines (MinXray, Northbrook, IL, USA) were integrated with qXR Version 3, a CE-certified and WHO-recommended AI software developed by Qure.ai (Mumbai, India). The software automatically analysed chest radiographs, generating a TB abnormality score ranging from 0.01 to 0.99, and also identified non-TB abnormalities. qXR Version 3 (Qure.ai Technologies Pvt. Ltd., Mumbai, India) is an AI-based computer-aided detection (CAD) software for automated interpretation of chest radiographs, developed using deep convolutional neural networks and trained on over four million chest radiographs and corresponding radiologist reports. For each radiograph, the software generates abnormality-specific probability scores and flags both TB-related and non-TB findings. The TB abnormality score reflects the model’s estimated probability that a radiograph contains TB-related findings, ranging from 0.01 to 0.99. Separate outputs simultaneously identify non-TB abnormalities, including cardiomegaly, pleural effusion, fibrosis, consolidation, reticulonodular patterns, and a blunted costophrenic angle, which formed the basis for CVD and CRD referral classification in this study. The software holds CE MDR Class IIb certification and has been independently validated against WHO Target Product Profile benchmarks for TB screening in adults. In this study, qXR Version 3 was deployed in offline mode, with image analysis completed in under one minute per radiograph. The model was applied using its pre-trained configuration and was not retrained or recalibrated on the study population; the implications of this are addressed in the limitations section.

#### Abnormal chest radiograph

A chest radiograph was classified as abnormal if the AI software localised any radiographic abnormalities, including TB-related abnormalities (e.g., cavities, nodules, consolidation) or non-TB abnormalities (e.g., cardiomegaly, fibrosis, pleural effusion, or structural changes), based on predefined classification outputs of the qXR algorithm.

#### Presumptive TB

Defined as the presence of TB-related symptoms (using the WHO four-symptom screening tool: cough, fever, weight loss, or night sweats) [18, 27] and/or an AI-generated qXR score ≥ 0.5. The 0.5 threshold is the manufacturer’s recommended default and was the standard threshold applied in AI-enabled TB screening programmes at the time this study was conducted (January 2023 to December 2024) [28]. A study of AI-enabled TB screening using the same MinXray-qXR v3 system in rural northeast Nigeria, a setting analogous to the current study, found that a threshold of 0.50 identified 89.4% of persons with bacteriologically confirmed TB with 62.8% specificity. This cut-off reduced the molecular testing burden by 42%, requiring only 435 tests compared to 738 tests at a 0.3 cut-off (which yielded a lower specificity of 29.8%) [29]. The more recent shift toward a lower threshold of 0.30 in some active case-finding programmes, aimed at maximising sensitivity, emerged largely after the design and initiation of this study. The 0.50 threshold was therefore selected on the basis of the evidence available at the time, to optimise specificity and manage downstream Xpert MTB/RIF cartridge and budgetary constraints in the study setting.

#### Non-TB abnormalities (CVDs and CRDs)

Radiographic abnormalities were categorised as suggestive of CVDs or CRDs based on structured outputs generated by the qXR algorithm. The AI software localises multiple findings simultaneously, enabling classification beyond TB. In collaboration with consultant cardiologists and pulmonologists at the partnering tertiary hospitals, a decision-support algorithm was developed before implementation that mapped each qXR non-TB output tag to a differential-diagnosis group and a corresponding referral pathway, drawing on established radiographic criteria for chronic lung and cardiac disease and on the recognised role of chest radiography in identifying cardiovascular and respiratory disease in low-resource settings [19, 20]. CVD-related findings included cardiomegaly, and cardiomegaly in combination with pleural effusion or a blunted costophrenic angle, consistent with features of cardiac failure; these were routed to a cardiologist irrespective of the AI TB score. CRD-related findings included pulmonary and pleural abnormalities such as fibrosis, consolidation, reticulonodular patterns, pleural effusion, and other chronic lung changes; these were routed to a pulmonologist where the TB pathway did not apply. Findings that were ambiguous or overlapping (for example, an abnormality carrying both a TB score ≥ 0.5 and a non-TB tag) were prioritised into the TB pathway first, with non-TB review arranged in parallel. Table 1 presents the full classification framework used. These classifications guided referral for further clinical evaluation and were not considered definitive diagnoses.

**Table 1:** AI-output classification and referral framework used in the INTEGRATE-TB study

Chest X-ray finding	Differential diagnoses considered	Referral pathway
Normal / no finding	Rule out multiple conditions	If TB symptoms present (cough, fever, weight loss, or night sweats): sputum collected for Xpert MTB/RIF testing.
TB-associated findings: radiological signs of TB, opacity, consolidation, cavity, fibrosis, hilar enlargement, nodule, pleural effusion, blunted costophrenic angle	TB, other lung infections, pneumoconiosis, pneumonia, chronic lung infections, healed/reactivated TB, lymphoma and other malignancies, lung cancer	If AI score ≥ 0.5: TB programme (sputum collection, Xpert MTB/RIF testing) — TB referral pathway. If AI score < 0.5 with abnormality present and not already classified as symptomatic presumptive TB: pulmonologist review — non-TB CRD pathway.
Cardiomegaly; cardiomegaly with pleural effusion or blunted costophrenic angle	Features of heart failure / cardiac failure	Cardiologist review — CVD pathway (irrespective of AI TB score).

*AI* artificial intelligence, *CRD* chronic respiratory disease, *CVD* cardiovascular disease, *TB* tuberculosis, *Xpert MTB/RIF* Xpert Mycobacterium tuberculosis/rifampicin assay

### Diagnostic and referral pathways

People with presumptive TB provided sputum samples on site, which were transported to nearby GeneXpert laboratories following WHO-recommended biosafety and triple-packaging protocols [24]. Sputum was tested using the Xpert MTB/RIF assay on the GeneXpert platform (Cepheid, Sunnyvale, CA, USA). People with bacteriologically confirmed TB were linked to treatment at the nearest DOTS facility or a facility preferred by the participant, in line with Nigeria’s national TB guidelines.

For individuals with TB-suggestive radiographic abnormalities identified by AI but with a negative Xpert MTB/RIF result or inability to produce sputum, chest radiographs and clinical histories were reviewed remotely by radiologists via a secure cloud-based platform. Based on this review, a clinical decision was made to initiate TB treatment or pursue alternative management in accordance with national TB guidelines and WHO diagnostic recommendations [18, 24].

Participants with non-TB abnormalities suggestive of CVDs or CRDs were referred to partnering tertiary hospitals: Federal Teaching Hospital Abakaliki (Ebonyi State) and Dalhatu Araf Specialist Hospital (Nasarawa State). Prior to implementation, the project team conducted advocacy engagements with the management and clinical departments of both facilities to establish an expedited referral pathway. Under this arrangement, referred participants bypassed routine General Outpatient Department (GOPD) procedures entirely. On arrival, participants presented their AI-generated screening report from the community outreach, and a nurse in the Internal Medicine Department opened a clinic folder and directed them immediately to the relevant specialist: consultant cardiologists for CVD-suggestive findings and consultant pulmonologists for CRD-suggestive findings. Fortuitously, the cardiology and respiratory clinic days at both facilities coincided, enabling joint scheduling of referral visits.

At the referral facilities, individuals with CVD-suggestive findings were assessed clinically and had their blood pressure measured. Where the individual could afford it, electrocardiography (ECG) and echocardiography were requested. Where these investigations were not affordable, as the project did not cover treatment costs, the consultant cardiologist based the diagnosis and treatment decision on clinical examination and blood pressure findings alone, and initiated antihypertensive or other appropriate treatment according to national clinical guidelines. For individuals with CRD-suggestive findings, the consultant pulmonologist performed clinical examination and requested spirometry and further respiratory investigations. Where individuals could not afford these investigations, as the project did not cover diagnostic costs, clinical assessment formed the basis for management decisions. Follow-up was coordinated through routine specialist outpatient clinic appointments at each facility.

To address the social and logistical barriers of travelling to an unfamiliar town for participants from hard-to-reach rural communities, a “batched referral” strategy was introduced. Community volunteers, who were already known and trusted by community members, coordinated groups of referred participants to travel together to the hospitals on scheduled clinic days, aligned with specialist clinic schedules. The arrangement ensured that participants were dropped directly at the hospital rather than at a bus park, reducing the navigation burden for individuals who had rarely or never visited the town. Travelling in a group with a familiar community volunteer also reduced the anxiety and unfamiliarity of the journey for people navigating an urban health facility for the first time. The project did not cover or subsidise transport fares, and participants were responsible for their own costs. For those who could not afford the journey, the referral could not be completed regardless of the coordination support offered, and the unaffordability of transport remained a significant barrier to referral completion.

### Fidelity, supervision, and quality assurance

Standard operating procedures and job aids guided all field activities. Field teams received initial and refresher training, supported by routine supervision. Digital data collection tools incorporated validation checks to enhance data quality, consistent with WHO recommendations for programme monitoring [26]. Weekly supervisory visits and monthly review meetings assessed protocol adherence and challenges. A dedicated messaging platform facilitated real-time troubleshooting, and monthly feedback was shared with stakeholders to support continuous quality improvement.

### Data collection and analysis

Data were collected using the electronic Q-Track platform provided with the AI model, and complementary paper-based registers capturing demographic characteristics, AI screening outputs, laboratory results, and referral outcomes. Paper records were routinely cross-checked against electronic data to ensure consistency.

Data were cleaned and analysed using Microsoft Excel. Descriptive statistics were used to summarise participant characteristics, screening outcomes, diagnostic yield, and linkage-to-care rates, consistent with recommended approaches for programmatic TB screening studies [27].

## Results

### Participant characteristics and screening volume

Between January 2023 and December 2024, a total of 9,585 individuals were screened using AI-integrated portable digital chest X-ray through 93 community-based outreach activities conducted across five LGAs in Ebonyi and Nasarawa States. Of those screened, 4,053 (42%) were male, and 5,532 (58%) were female.

Screening volume ranged from 80 to 120 individuals per outreach day, with variations influenced by weather conditions and community mobilisation. Communities with larger populations or higher screening yields were prioritised for repeat outreach visits. Screening volume and outcomes disaggregated by LGA are presented in Table 2.

**Table 2:** Characteristics of participants screened in community outreach activities

State	LGA	Total screened	Male *n* (%)	Female *n* (%)
Ebonyi	Ikwo	2,432	914 (38%)	1,518 (62%)
	Ishielu	2,616	819 (31%)	1,797 (69%)
	Izzi	1,727	774 (45%)	953 (55%)
**Ebonyi subtotal**		**6**,**775 (71%)**	**2**,**507 (37%)**	**4**,**268 (63%)**
Nasarawa	Karu	1,587	886 (56%)	701 (44%)
	Lafia	1,223	662 (54%)	561 (46%)
**Nasarawa subtotal**		**2**,**810 (29%)**	**1**,**548 (55%)**	**1**,**262 (45%)**
**Total**		**9**,**585 (100%)**	**4**,**053 (42%)**	**5**,**532 (58%)**

Median age 41 years (range 6–101 years)

Column percentages for subtotals and total are shown against the overall number screened; the Male and Female percentages are row percentages within each area

*LGA* Local Government Area

### Radiographic findings and AI outputs

Of the 9,585 chest radiographs acquired during community outreach activities, 6,419 (67%) were classified as normal, while 3,166 (33%) were flagged as abnormal by the AI software. Here, an abnormal radiograph is defined as one in which the AI software identified any radiographic abnormality, whether TB-related or non-TB (CVD- or CRD-suggestive). Among individuals with normal radiographs, 2,958 (46%) were male, and 3,461 (54%) were female. Of those with abnormal radiographs, 1,095 (35%) were male, and 2,071 (65%) were female.

The proportion of abnormal radiographs was higher in Ebonyi State (35%) compared with Nasarawa State (29%). Abnormality rates further disaggregated by sex and LGA are presented in Table 3.

**Table 3:** Abnormalities suggestive of CVD, CRD, and presumptive TB by LGA and sex

State / LGA	Abn. Male *n*	Abn. Female *n*	Abn. Total *n*	CVD Male *n* (%)	CVD Female *n* (%)	CRD Male *n* (%)	CRD Female *n* (%)	Presump. TB ≥ 0.5 Male *n* (%)	Presump. TB ≥ 0.5 Female *n* (%)
Ebonyi – Ikwo	270	538	808	39 (14%)	219 (41%)	110 (41%)	209 (39%)	121 (45%)	110 (20%)
Ebonyi – Ishielu	247	679	926	45 (18%)	264 (39%)	111 (45%)	279 (41%)	91 (37%)	136 (20%)
Ebonyi – Izzi	248	360	608	32 (13%)	84 (23%)	134 (54%)	228 (63%)	80 (32%)	50 (14%)
**Ebonyi subtotal**	**765**	**1**,**577**	**2**,**342**	**116 (15%)**	**567 (36%)**	**355 (46%)**	**716 (45%)**	**292 (38%)**	**296 (19%)**
Nasarawa – Karu	197	280	477	28 (14%)	61 (22%)	95 (48%)	160 (57%)	74 (38%)	59 (21%)
Nasarawa – Lafia	133	214	347	22 (17%)	58 (27%)	65 (49%)	123 (58%)	46 (34%)	33 (15%)
**Nasarawa subtotal**	**330**	**494**	**824**	**50 (15%)**	**119 (24%)**	**160 (49%)**	**283 (57%)**	**120 (36%)**	**92 (19%)**
**Total**	**1**,**095**	**2**,**071**	**3**,**166**	**166 (15%)**	**686 (33%)**	**515 (47%)**	**999 (48%)**	**412 (38%)**	**388 (19%)**

Percentages are row percentages by sex within each abnormality category

*Abn.* abnormal radiograph (TB or non-TB combined), *CRD* chronic respiratory disease, *CVD* cardiovascular disease, *LGA* Local Government Area, *TB* tuberculosis

### Tuberculosis screening, diagnosis, and linkage to care

A total of 1,336 (14%) individuals were classified as people with presumptive TB based on the presence of TB-related symptoms, an AI-generated qXR score ≥ 0.5, or both. Of these, 800 (60%) individuals were identified based on an AI score ≥ 0.5, while 536 (40%) individuals were identified based on symptoms alone with qXR scores < 0.5. Overall, people with presumptive TB comprised 694 (52%) males and 642 (48%) females. Among people with presumptive TB, 1,123 individuals (84%) were able to produce spot sputum samples. Testing with the Xpert MTB/RIF assay identified 204 individuals with bacteriologically confirmed TB, representing 18% of those tested. Of these people with confirmed TB, 124 (61%) were male, and 80 (39%) were female.

Linkage-to-care data showed that 194 (95%) of people with bacteriologically confirmed TB were successfully initiated on treatment at a DOTS facility, either at the nearest facility or at a facility preferred by the patient. In addition, 199 individuals with TB-suggestive radiographic abnormalities but a negative Xpert MTB/RIF result or inability to produce sputum were clinically diagnosed and initiated on TB treatment following radiologist review of chest radiographs and/or clinical assessment. The persons with clinically diagnosed TB include 114 males and 85 females.

### Non-TB abnormalities and referral outcomes

**Fig. 1:**
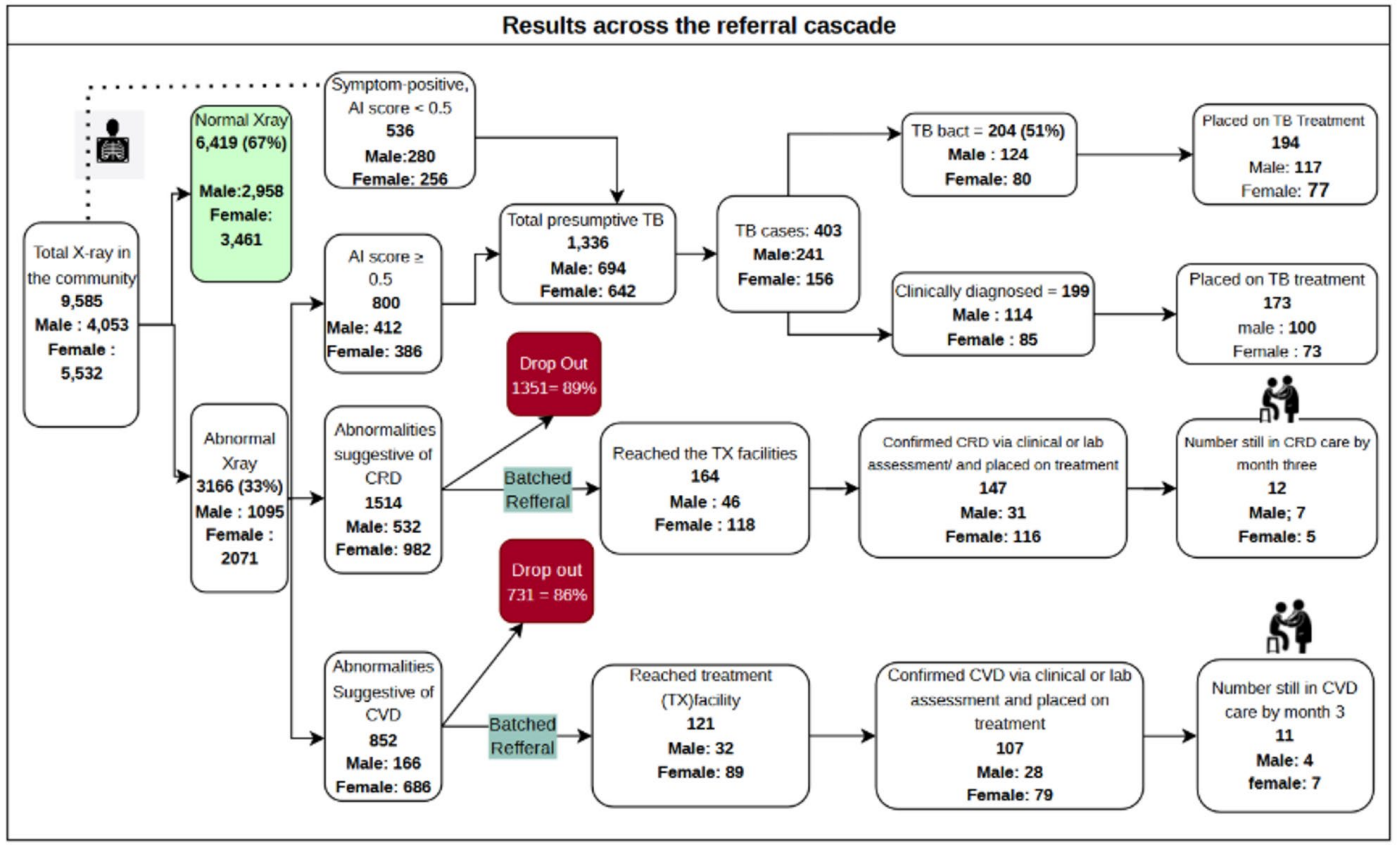
Results across the referral cascade for tuberculosis, cardiovascular disease, and chronic respiratory disease, INTEGRATE-TB project, Ebonyi and Nasarawa States, Nigeria, January 2023–December 2024. CRD, chronic respiratory disease; CVD, cardiovascular disease; TB, tuberculosis; Xpert MTB/RIF, Xpert Mycobacterium tuberculosis/rifampicin assay

Among the 3,166 abnormal chest radiographs, the AI software identified 2,367 individuals (75%) with abnormalities suggestive of non-TB conditions, including CVDs and CRDs. Specifically, 852 individuals had radiographic findings suggestive of CVDs, and 1,514 individuals had findings suggestive of CRDs. All individuals with non-TB abnormalities were referred to partnering tertiary hospitals for further clinical or laboratory evaluation. Of those referred, 121 individuals with radiographic abnormalities suggestive of CVD and 164 with radiographic abnormalities suggestive of CRD reached the referral facility. On reaching the facility, clinical or laboratory assessment confirmed 107 of the 121 CVD and 147 of the 164 CRD diagnoses, and all confirmed individuals were initiated on treatment at that same visit in accordance with national clinical guidelines. At three-month follow-up, 11 of those initiated on CVD treatment and 12 of those initiated on CRD treatment were documented as still engaged in care. The full CVD and CRD referral cascade are shown in Fig. 1.

### Sex patterns across the cascade

Of the 9,585 individuals screened, 5,532 (58%) were female, and 4,053 (42%) were male. The pattern of female participation differed by state: in Ebonyi State, females accounted for 63% of those screened (4,268 of 6,775), while in Nasarawa State, males accounted for 55% of those screened (1,548 of 2,810). Among the 3,166 individuals with abnormal radiographs, 2,071 (65%) were female and 1,095 (35%) were male.

Despite higher female participation overall, males accounted for the majority of people with presumptive TB, 694 of 1,336 (52%), and of those with bacteriologically confirmed TB, 124 of 204 (61%) were male. Among the 199 people clinically diagnosed with TB, 114 (57%) were male.

In contrast, females accounted for the majority of individuals with radiographic abnormalities suggestive of CVD and CRD. Of the 852 individuals with CVD-suggestive abnormalities, 686 (81%) were female. Of the 1,514 with CRD-suggestive abnormalities, 982 (65%) were female.

## Discussion

This study demonstrates that community-based AI-enabled chest X-ray screening is a feasible platform for integrated detection of TB, CVDs, and CRDs in underserved rural communities in Nigeria. Among the 9,585 individuals screened, 3,166 (33%) chest radiographs were flagged as abnormal by the AI system, indicating a substantial number of radiographic abnormalities requiring further clinical evaluation that may otherwise remain undetected within routine primary care [30, 31]. The findings provide insights into the distribution of abnormalities, sex differences in screening participation and disease patterns, and the emerging burden of NCDs in rural LMIC settings. However, considerable attrition along referral pathways for non-TB conditions was observed. Although individuals with NCD-related abnormalities were referred for further evaluation, only 12% completed referral, that is, reaching the treatment facility for further evaluation and care; this reflects structural gaps in service availability. These findings highlight both the public health value of multi-disease community screening and the importance of strengthening referral systems and decentralised services to ensure that early detection translates into effective care [32, 33].

A key finding of this study is the high proportion of abnormal chest radiographs detected during community screening. Approximately one-third (33%) of all screened individuals had abnormal chest radiographs, indicating substantial unmet diagnostic need in these rural communities. Similar abnormality rates have been reported in community-based chest X-ray screening initiatives in other high TB burden settings and are often linked to delayed health-seeking behaviour, limited access to diagnostic services, and prolonged exposure to environmental and occupational risk factors [34, 35].

Chest radiography remains one of the most effective population-level tools for detecting pulmonary disease, including TB and other cardiopulmonary conditions [18]. The integration of computer-aided detection (CAD) systems has further improved the feasibility of large-scale screening in resource-limited settings by enabling rapid interpretation and reducing reliance on specialist radiologists. Several studies have demonstrated that AI-based CAD systems can achieve diagnostic performance comparable to trained human readers for TB screening and are increasingly recommended for use in high-burden settings [16, 36, 37]. In communities with limited access to routine diagnostic services, AI-supported chest radiography therefore offers an effective triage tool for identifying both TB and other pulmonary abnormalities. The high abnormality yield observed in this study supports the rationale for integrated screening models that address multiple cardiopulmonary conditions rather than focusing solely on TB [30, 38].

The sex patterns observed across screening and detection likely reflect a combination of biological, behavioural, and contextual factors. The higher female participation overall, and the regional contrast between Ebonyi and Nasarawa, is consistent with evidence that women in southern Nigeria often engage more actively in community-based preventive services, while participation among women in northern and north-central settings can be constrained by mobility and household decision-making dynamics [39, 40]. The higher burden of presumptive and confirmed TB among men is consistent with global epidemiological evidence and has been attributed to occupational exposure, tobacco and alcohol use, and delayed care-seeking [5, 41]. The predominance of women among those with abnormalities suggestive of CVD or CRD may reflect gendered environmental exposures, including household air pollution from biomass fuel use, as well as lower rates of routine cardiovascular screening among women in many LMICs [33, 42, 43]. These patterns underline the value of gender-sensitive strategies within integrated screening programmes.

Another important finding of this study is the substantial burden of NCD-related abnormalities detected during screening. Among the 3,166 abnormal radiographs, approximately 2,367 (75%) showed features suggestive of CVDs or CRDs, indicating that the majority of abnormalities identified were related to non-TB conditions. These findings challenge the perception that NCDs are primarily urban health problems and instead highlight the ongoing epidemiological transition affecting rural populations in LMICs [44]. Evidence from Nigeria and other sub-Saharan African countries has documented increasing prevalence of hypertension, cardiovascular risk factors, and chronic respiratory disease in rural communities [7]. Environmental exposures such as biomass fuel use, alongside changing lifestyles and ageing populations, are contributing to this growing burden [44]. Integrated screening initiatives such as INTEGRATE-TB therefore provide valuable community-level data that can inform health system planning and support the decentralisation of NCD services within primary healthcare platforms. Without such integrated approaches, many of these conditions would likely remain undiagnosed until advanced stages of disease [30].

The contrasting linkage outcomes for TB and non-TB abnormalities highlight important differences in health system capacity. The 95% treatment initiation rate among people with bacteriologically confirmed TB reflects the relative maturity of Nigeria’s TB programme, in which services are decentralised, free at the point of care, and embedded within primary healthcare facilities [5]. In contrast, linkage to care for individuals with abnormalities suggestive of CVD or CRD was substantially lower, with only 12% completing referral. While the batched referral strategy reduced the social and logistical barriers of travel for participants from hard-to-reach communities, the project did not cover or subsidise transport fares, and for those who could not afford the journey, the referral could not be completed, regardless of the coordination support offered. Transport unaffordability therefore remained a significant and independent financial barrier to referral completion, distinct from the structural absence of decentralised NCD services. This gap reflects systemic constraints rather than limitations of the screening approach itself: in many rural Nigerian settings, CVD and CRD services are unavailable at the primary care level and require referral to higher-level facilities, introducing barriers related to travel distance, transport costs, and out-of-pocket expenditure [33]. Detection alone does not guarantee access to treatment when health systems lack the capacity to manage identified conditions. The WHO Package of Essential NCD Interventions recommends decentralising basic NCD services to primary care, including hypertension screening, diabetes testing, and access to essential medicines [43]; integrating such services within existing TB platforms could reduce fragmentation and improve continuity of care.

These findings can be situated within a broader shift toward point-of-care imaging in low- and middle-income country health systems. Portable, AI-enabled chest X-ray deployed at community outreach sites represents a shift away from facility-bound, specialist-dependent diagnostics toward accessible, context-appropriate tools [19, 45]. By bringing diagnostic capacity directly into hard-to-reach communities, these tools support a model of care in which the first point of meaningful clinical contact is in the community rather than in a health facility. This is particularly significant in settings where tertiary facilities are geographically and financially inaccessible to most of the population. Framing community chest radiography as point-of-care imaging helps explain both its strength, the ability to detect cardiopulmonary abnormalities at scale without on-site radiologists, and its limitation, that imaging access alone cannot substitute for the downstream services required to confirm and manage non-communicable disease. This perspective reinforces the argument that diagnostic innovation must be paired with investment in primary care capacity to translate early detection into improved health outcomes.

A small proportion of pre-treatment loss to follow-up occurred among individuals diagnosed with TB. Of the 204 people with bacteriologically confirmed TB, approximately 5% were not initiated on treatment, reflecting the challenges of tracking individuals identified through community outreach activities, particularly in large congregate settings such as markets. Similar patterns have been observed in other community-based TB case-finding programmes [35]. To mitigate this risk, the programme engaged trusted community volunteers to support participant tracking, communicate results, and facilitate referral and treatment initiation. Community-led approaches have been shown to improve linkage to care by leveraging local trust and social networks and are recommended in WHO guidance on community-based TB care [24].

The findings suggest that integrated screening programmes will be most effective when aligned with accessible treatment services. TB screening platforms can serve as entry points for broader cardiopulmonary health assessment, but meaningful improvements in NCD outcomes will require strengthening primary healthcare capacity to deliver basic services such as blood pressure measurement, chronic respiratory assessment, and initial treatment [43]. Combining AI-enabled chest X-ray screening with complementary low-cost diagnostic tools could improve early identification of cardiovascular risk while reducing unnecessary referrals. Sustained community engagement, structured referral systems, and the involvement of trusted community volunteers should remain central components of integrated outreach models.

A major strength of this study is its implementation under routine programme conditions in rural communities, generating operational evidence on the feasibility of AI-enabled integrated screening. Standardised screening protocols, digital data systems, and ongoing supervision supported implementation fidelity and data quality. Several limitations should be considered. First, participation was voluntary, and outreach was free, which may have introduced selection bias: individuals with symptoms may have been more likely to attend, potentially inflating the observed proportions of abnormal radiographs and TB yield. Second, verification bias is likely, as not all abnormal radiographs received confirmatory testing, particularly in the non-TB pathway; the true proportion of confirmed CVD or CRD among those with abnormal AI outputs therefore cannot be precisely estimated. Third, follow-up data were captured only through designated referral facilities, so referral completion and continuity of care for non-TB conditions may be underestimated if some participants sought care elsewhere. Fourth, HIV status was not collected during community outreach activities, which limited stratified interpretation of screening yield by this risk factor. Prior TB history was collected selectively for individuals who proceeded to radiologist review following a negative or inconclusive Xpert MTB/RIF result, where it informed the clinical diagnosis decision; however, it was not systematically recorded across all screened participants. Finally, imaging artefacts caused by patterned clothing produced false-positive readings during early implementation, although mitigation measures were subsequently introduced. We recommend that future integrated screening programmes collect HIV status, prior TB history, and relevant NCD risk factors prospectively to support more detailed analysis.

## Conclusions

Community-based, AI-enabled chest X-ray screening is feasible for TB case finding in rural Nigeria and achieves high linkage to TB treatment. The same platform identified a substantial number of radiographic abnormalities suggestive of cardiovascular and chronic respiratory disease, confirming that a single community screening contact can generate value beyond TB. However, most non-TB abnormalities were not confirmed or managed, because referral completion was constrained by transport costs and by the limited decentralisation of NCD services. Integrated screening therefore delivers on early detection but not yet on continuity of care for non-communicable conditions. To close this gap, AI-enabled chest X-ray screening should be paired with strengthened primary healthcare capacity, complementary low-cost diagnostics such as blood pressure measurement, decentralised NCD services, and context-specific community engagement and referral support. These measures would allow integrated screening to translate detection into improved health outcomes for underserved rural populations.

## Data Availability

The de-identified dataset generated and analysed during the current study is available in the Figshare repository: 10.6084/m9.figshare.32687448. No custom code was generated, as all analyses were descriptive and conducted in Microsoft Excel.

## Abbreviations

AI: Artificial Intelligence
CAD: Computer-Aided Detection
CCFN: Catholic Caritas Foundation of Nigeria
CE MDR: Conformité Européenne Medical Device Regulation
CRD: Chronic Respiratory Disease
CVD: Cardiovascular Disease
DOTS: Directly Observed Treatment, Short-course
ECG: Electrocardiography
GOPD: General Outpatient Department
LGA: Local Government Area
LGTBLS: Local Government TB and Leprosy Supervisor
LMIC: Low- and Middle-Income Country
MTB/RIF: Mycobacterium tuberculosis / Rifampicin
NCD: Non-Communicable Disease
NHREC: National Health Research Ethics Committee
PDX: Portable Digital X-ray
PEN: Package of Essential NCD Interventions
TB: Tuberculosis
TIDieR: Template for Intervention Description and Replication
WHO: World Health Organization
